# RNA binding protein 24 deletion disrupts global alternative splicing and causes dilated cardiomyopathy

**DOI:** 10.1007/s13238-018-0578-8

**Published:** 2018-09-28

**Authors:** Jing Liu, Xu Kong, Mengkai Zhang, Xiao Yang, Xiuqin Xu

**Affiliations:** 1 The Institute of Stem Cell and Regenerative Medicine, Medical College, Xiamen University, Xiamen, 361100 China; 2 State Key Laboratory of Proteomics, Genetic Laboratory of Development and Disease, Institute of Biotechnology, Beijing, 100071 China

**Keywords:** RNA binding protein, RBM24, dilated cardiomyopathy, alternative splicing, heart failure

## Abstract

RNA splicing contributes to a broad spectrum of post-transcriptional gene regulation during normal development, as well as pathological manifestation of heart diseases. However, the functional role and regulation of splicing in heart failure remain poorly understood. RNA binding protein (RBP), a major component of the splicing machinery, is a critical factor in this process. RNA binding motif protein 24 (RBM24) is a tissue-specific RBP which is highly expressed in human and mouse heart. Previous studies demonstrated the functional role of RBM24 in the embryonic heart development. However, the role of RBM24 in postnatal heart development and heart disease has not been investigated. In this paper, using conditional RBM24 knockout mice, we demonstrated that ablation of RBM24 in postnatal heart led to rapidly progressive dilated cardiomyopathy (DCM), heart failure, and postnatal lethality. Global splicing profiling revealed that RBM24 regulated a network of genes related to cardiac function and diseases. Knockout of RBM24 resulted in misregulation of these splicing transitions which contributed to the subsequent development of cardiomyopathy. Notably, our analysis identified RBM24 as a splice factor that determined the splicing switch of a subset of genes in the sacomeric Z-disc complex, including Titin, the major disease gene of DCM and heart failure. Together, this study identifies regulation of RNA splicing by RBM24 as a potent player in remodeling of heart during postnatal development, and provides novel mechanistic insights to the pathogenesis of DCM.

**Electronic supplementary material:**

The online version of this article (10.1007/s13238-018-0578-8) contains supplementary material, which is available to authorized users.

## Introduction

Mammalian genomes encode for a large number of RNA binding proteins (RBPs), which play an important role on post-transcriptional regulation occurring during development and disease. RBPs are responsible for controlling tissue-specific gene expression by regulating alternative splicing (AS). AS regulation differentially produces structurally and functionally distinct messenger RNA (mRNA) and protein isoforms, thus provides a powerful additional mechanism with which to control cell fate and physiological response (Cooper, 2005; Kalsotra and Cooper, 2011). Misregulated AS events have a significant impact in many human diseases (Hallegger et al., 2010).

Cardiomyopathy ranks among the most prevalent causes of premature death in the world. Among these primary cardiomyopathies, the most common form is dilated cardiomyopathy (DCM) (Tayal et al., 2017). In DCM, the heart becomes weakened, enlarged, and unable to pump blood efficiently. Although DCM causative mutations have been detected in almost 50 genes so far, most of the genetic studies are focused on genes involved in the sarcomere organization that are important for heart contraction, such as *TTN*, *TPM1*, *MYH7* and *TNNT2* (Tayal et al., 2017).

Recent studies provided new insights into the pathology of DCM. It is found alternations in AS were associated with heart diseases in mouse models and human (Kong et al., 2010; Lara-Pezzi et al., 2013). Individuals with ischemic cardiomyopathy, DCM, or aortic stenosis showed strong changes in AS of key sarcomeric genes such as *TNNT2*, *TNNI3*, *MYH7* and *TTN* (Kong et al., 2010; Roberts et al., 2015). Genes encoding ion channels (*SCN5A, CAMK2D* and *Ryr*) and signaling molecules (*Tbx3*, *Tbx5* and *Tbx20*) were also regulated by AS in some cardiac defects (Lara-Pezzi et al., 2013). These data strongly support the hypothesis that a component of the splicing machinery, RBP, is a critical factor in cardiomyopathy.

In fact, heart-specific knockout mice have shown that some RBPs knockout could dramatically alter the splicing of cardiac proteins and result in heart diseases, such as MBNL1 (LeMasters et al., 2012), RBFOX1 (Gao et al., 2016) and RBFOX2 (Wei et al., 2015). These studies provide new insights into a hitherto under-appreciated form of DCM. However, only *RBM20* mutations have been discovered in individuals with early-onset familial DCM so far (Wells et al., 2013; Beqqali et al., 2016). Thus, our knowledge regarding the role of RBPs on DCM is still limited.

We have previously reported that RNA binding motif protein 24 (RBM24) was enriched in human embryonic stem cells (ESCs)-derived cardiomyocytes and highly expressed in human and mouse heart (Xu et al., 2008; Xu et al., 2009). We further characterized the functional role of RBM24 in the heart development in zebrafish model (Poon et al., 2012). Our recent study identified RBM24 as a key splicing regulator that promoted cardiac differentiation of ESCs (Zhang et al., 2016). Yang et al. reported that RBM24 null mice died from multiple cardiac malfunctions (Yang et al., 2014). These findings revealed the involvement of RBM24 at the early stage of heart development, including the establishment of specific cardiac cell types and cardiac morphogenesis. Although the fundamental requirement for RBM24 in earlier heart development is well established, the role of RBM24 in late heart development and how RBM24 controls aspects of postnatal heart development remains to be explored. More importantly, its potential contribution to disease remains unclear.

Previous study suggested that RBM24 null mice were embryonic lethal (Yang et al., 2014). To circumvent this issue, we used a Cre-loxP approach to obtain conditional RBM24 knockout mice. Here, we report that cardiac-specific ablation of RBM24 at postnatal stage leads to rapidly progressive DCM and heart failure. Our global analysis discovered a large number of splicing switches which were undefined in previous studies focusing on early cardiogenesis. Therefore, our study reveals for the first time that RBM24-dependent RNA splicing is an important post-transcriptional regulatory circuit in postnatal heart, which has a significant role in the pathogenesis of DCM. Importantly, our study offers a global view about how an RBP functions as a master regulator of a broad network of RNA isoform switches, and thereby extends the emerging concept that DCM can be triggered by dysfunctional RNA processing.

## RESULTS

### Generation of cardiac-specific RBM24 conditional knockout mice

**Figure 1:**
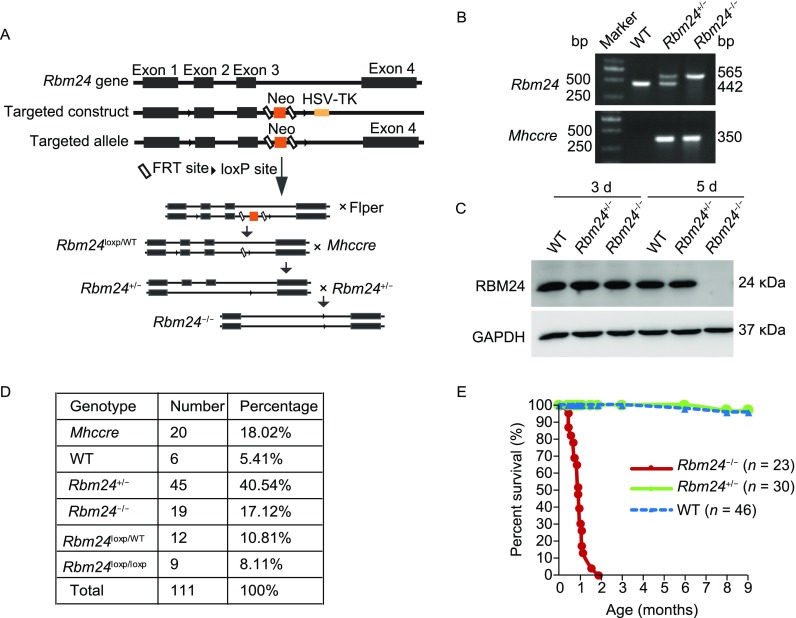
**Generation of cardiac-specific RBM24 knockout mice**. (A) Cre-loxP system was used to disrupt the RBM24 tissue-specifically in the heart. Schematic diagram represents WT and mutant loci of *Rbm24* gene together with the targeting vector. Exons for the gene encoding *Rbm24* were represented by a black box. We generated conditional RBM24 knockout mice by cross-breeding *Rbm24*^loxp/WT^ mice with *Mhc*cre knockin mice. (B) PCR genotyping analysis of RBM24 knockout mice. *Rbm24*^−/−^ produced the expected 565 bp mutant fragment compared with a 442 bp wild-type fragment. Data in each group are representative of 20–46 mice. (C) Western blot analysis of RBM24 protein from WT, *Rbm24*^−/−^ or *Rbm24*^+/−^ mice at postnatal day 3 and day 5. GAPDH served as a loading control. Data in each group are representative of 5–10 mice. (D) Viable mice were born at approximately Mendelian ratios. (E) Cumulative survival curve of *Rbm24*^−/−^, *Rbm24*^+/−^ and WT mice. See also Fig. S1

*RBM24* is expressed from embryonic cardiac development throughout adulthood (Poon et al., 2012). However, constitutive knockout mice for RBM24 are embryonic lethal (Yang et al., 2014), precluding assessment of the function of RBM24 after cardiogenesis. To understand the functional involvement of RBM24 in postnatal heart and potential contribution to heart diseases, we generated a conditional deletion allele of the mouse *Rbm24* gene by homologous recombination in mouse ESCs. Two loxP sites were engineered to flank a region of *Rbm24* genomic DNA encoding exons 2–3 (Fig. 1A). Mice carrying conditional *Rbm24* allele were crossed with mice carrying the Cre recombinase gene driven by the α-*Mhc* promoter (Fig. 1A), which expresses Cre recombinase in heart after birth (Wang et al., 2005). Cre-mediated recombination deleted *Rbm24* exon 2 and 3 between the loxP sites. This recombination was confirmed in the DNA of the mutant mice. As shown in Figure 1B, homozygous *Rbm24* mutant (*Rbm24*^−/−^) was given the expected 565 bp mutant fragment compared with a 442 bp wild-type (WT) fragment. Western blot (Fig. 1C) showed that RBM24 protein disappeared at 5 days old. Of note, RBM24 expression was not affected in heterozygous mice (*Rbm24*^+/−^) at the several postnatal stages we examined (Figs. 1C and S1).

### Targeted deletion of the RBM24 in postnatal heart leads to lethality

The *Rbm24*^−/−^ mice were born at the expected Mendelian ratio (Fig. 1D) and were externally indistinguishable from WT. However, knockout mice had some specific manifestations or behavioral changes as age increased, including weight loss, smaller body size, and shortness of breath (Videos S1–2). We noted *Rbm24*^−/−^ mice were suffering death, earliest at 11 days of age and all died before 2 months of age (Fig. 1E), when compared with survival of WT and *Rbm24*^+/−^ littermates. Preceding death, *Rbm24*^−/−^ mice became very fragile and exhibited decreased spontaneous activity. We observed congestion in the left ventricle and left atrium of *Rbm24*^−/−^ hearts after death, indicating *Rbm24*^−/−^ hearts suffered from impaired cardiac function. These results gave rise to a general implication that RBM24 protein performed fundamental function crucial for postnatal heart.

### RBM24 knockout mice exhibit dilated cardiomyopathy

**Figure 2:**
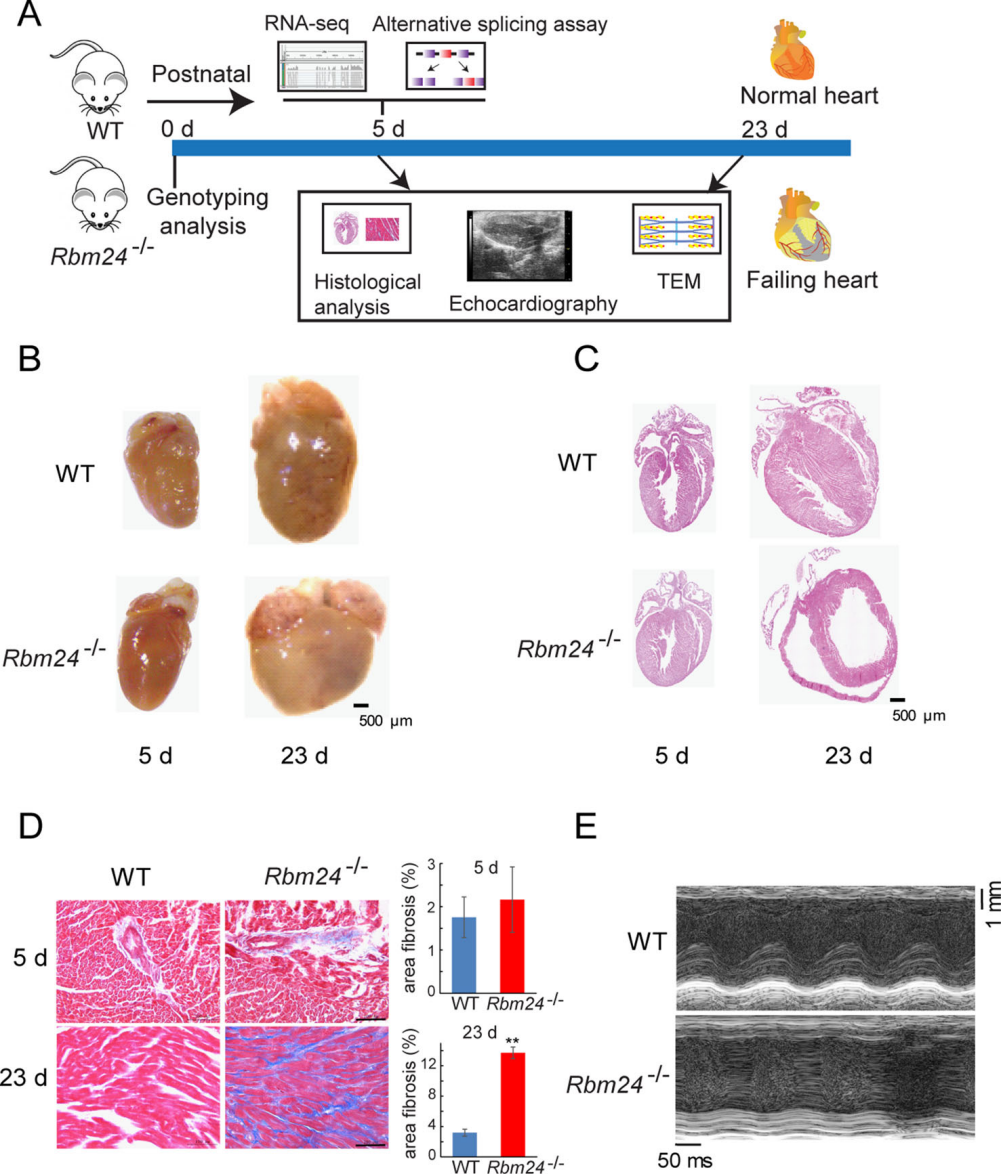
**Cardiac-specific RBM24 deletion leads to DCM**. (A) Schematic overview of experimental strategy. Assays performed at different time points as described in the Results section are indicated in the diagram. (B) Representative gross morphology of WT and *Rbm24*^−/−^ hearts of mice. Scale bar: 500 μm. (C) Representative cross-sectional images of hematoxylin & eosin staining of hearts from WT and *Rbm24*^−/−^ mice. Scale bar: 500 μm. (D) Left: Representative Masson’s trichrome staining of WT and *Rbm24*^−/−^ mouse heart sections. Scale bar: 100 μm. Right: Quantification of fibrotic area in heart sections displayed on the left, % area fibrosis = the sum of fibrotic area in heart/the sum of myocardial area in heart. The data were expressed as means ± SEM. **P < 0.01. Data are representative from 5–7 mice (B–D). (E) Examples of cardiac echocardiography of WT and *Rbm24*^−/−^ mice at postnatal day 25. Scale bar: 1 mm. See also Videos S1–2

To examine the pathology in detail, we next evaluated heart performance by echocardiography and cardiac histopathology on *Rbm24*^−/−^ and WT mice at postnatal day 5 (immediatedly after RBM24 deletion) and day 23 (*Rbm24*^−/−^ mice became very fragile) respectively. A schematic diagram of the experimental strategy is illustrated in Fig. 2A. At day 5, *Rbm24*^−/−^ mice did not exhibit morphological alternation (Fig. 2B–D, 5 d), and the cardiac function was normal (Table 1, 5 d). However, we observed increased dilation of the ventricle chambers, increased fibrosis, and decreased cardiac function of *Rbm24*^−/−^ hearts overtime (Figs. 2B-D and Table 1). By day 23, *Rbm24*^−/−^ mice exhibited DCM both in echocardiography (Table 1, 23 d) and cardiac histopathology (Fig. 2C, 23 d) examination, showing typical enlarged left ventricular chamber size and significant wall thinning. Both interventricular septal thickness at end systole (IVSs) and left ventricular posterior wall thickness at end systole (LVPWs) were markedly decreased in *Rbm24*^−/−^ mice (Table 1, 23 d). The changes in cardiac morphologies in *Rbm24*^−/−^ mice were accompanied by a dramatic decrease in left ventricular systolic function, shown by a significant decrease in fractional shortening (FS) and ejection fraction (EF) (Table 1, 23 d). Fig. 2E showed a representative image of the echocardiography study for *Rbm24*^−/−^ hearts, where a dramatic increase of left ventricular internal diameter (LVID) during both systole and diastole was observed. These data demonstrated that cardiac-specific RBM24 knockout mice developed typical DCM and heart failure (Table 1).

**Table 1:** Analysis of *in vivo* cardiac size and function by echocardiography in WT, *Rbm24*^+/−^ and *Rbm24*^−/−^ mice.

	Geno-types	IVSd (mm)	IVSs (mm)	LVIDd (mm)	LVIDs (mm)	LVPWd (mm)	LVPWs (mm)	EF (%)	FS (%)	LV Vold (μL)	LV Vols (μL)
5 d	WT (*n* = 6)	0.38 ± 0.02	0.70 ± 0.03	1.66 ± 0.11	0.82 ± 0.06	0.42 ± 0.02	0.65 ± 0.03	84.66 ± 0.82	50.51 ± 0.90	8.33 ± 1.25	1.30 ± 0.21
	*Rbm24*^−/−^ (*n* = 7)	0.38 ± 0.01	0.61 ± 0.02	1.71 ± 0.11	0.87 ± 0.08	0.43 ± 0.02	0.66 ± 0.02	83.42 ± 2.22	49.78 ± 2.39	9.02 ± 1.38	1.60 ± 0.34
	*P* value	0.992	0.061	0.771	0.687	0.759	0.798	0.657	0.808	0.741	0.514
23 d	WT (*n* = 7)	0.62 ± 0.06	0.99 ± 0.06	3.16 ± 0.10	1.82 ± 0.15	0.49 ± 0.04	0.94 ± 0.06	73.91 ± 3.53	42.67 ± 3.47	40.18 ± 3.03	10.93 ± 1.81
	*Rbm24*^+/−^ (*n* = 6)	0.61 ± 0.08	0.93 ± 0.10	3.17 ± 0.15	1.96 ± 0.15	0.53 ± 0.02	0.88 ± 0.05	69.33 ± 2.78	36.25 ± 3.10	41.07 ± 0.88	12.92 ± 2.22
	*Rbm24*^−/−^ (*n* = 9)	0.56 ± 0.03	0.74 ± 0.04	3.75 ± 0.20	3.11 ± 0.22	0.52 ± 0.03	0.69 ± 0.07	36.34 ± 4.49	17.42 ± 2.34	62.44 ± 7.85	40.92 ± 7.26
	*P* value (WT vs. *Rbm24*^−/−^)	0.416	0.007**	* 0.044	0.001***	0.575	* 0.027	<0.001***	<0.001***	* 0.042	** 0.005

Echocardiography was performed in the same nest mice. Note that mice at 5 days old were not anesthetized, while mice at 23 days old were anesthetized with isoflurane gas before echocardiography. IVSd, interventricular septal thickness at end diastole; IVSs, interventricular septal thickness at end systole; LVIDd, left ventricular internal diameter at end diastole; LVIDs, left ventricular internal diameter at end systole; LVPWd, left ventricular posterior wall thickness at end diastole; LVPWs, left ventricular posterior wall thickness at end systole; EF, ejection fraction; FS, fractional shortening; LV Vold, left ventricular volume at end diastole; LV Vols, left ventricular volume at end systole. Data were expressed as mean ± SEM. Significant differences between groups were determined by Student’s *t*-test. *P < 0.05, **P < 0.01, ***P ≤ 0.001

### RBM24-regulated AS changes underlie specific functional defects in postnatal *Rbm24*^−/−^ mice

We next aimed to elucidate the mechanism underlying the observed functional defects and pathological phenotypes by profiling the splicing program perturbed in day 5 *Rbm24*^−/−^ heart, the earliest time point after RBM24 deletion.

**Figure 3:**
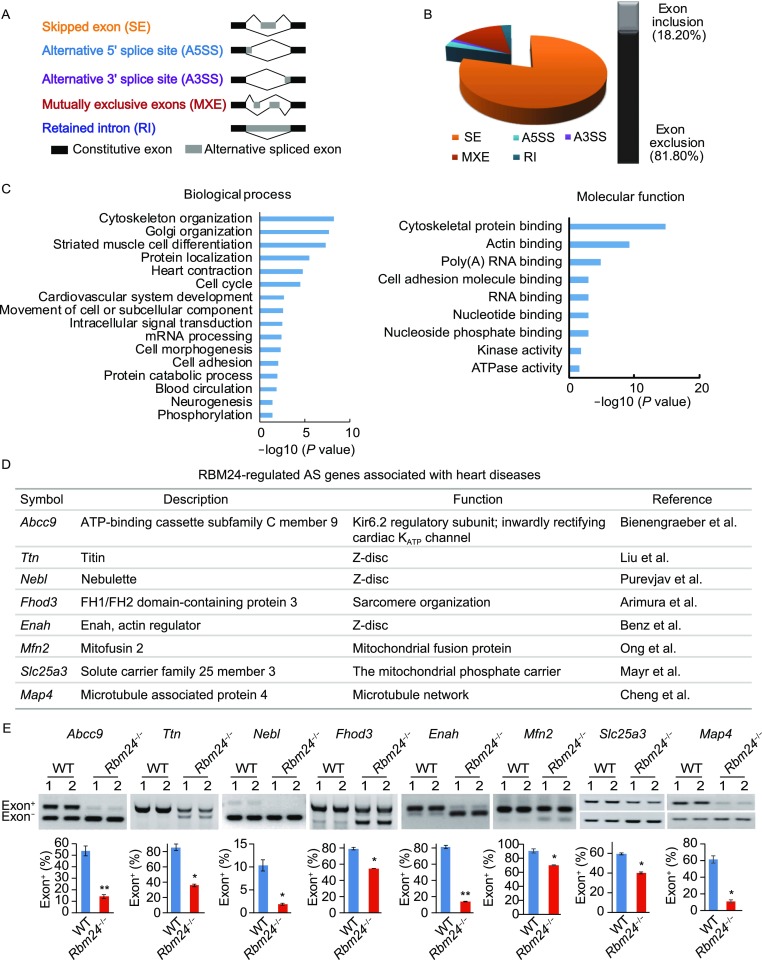
**RBM24 is a key splicing regulator important for postnatal heart function and disease**. (A) Types of AS patterns regulated by RBM24. (B) The proportion of AS types in response to RBM24 ablation. (C) Gene ontology analysis of RBM24-regulated AS events (biological process and molecular function). (D) RBM24-regulated AS genes associated with heart diseases. (E) Splicing analysis for RNAs related to cardiac diseases. RT-PCR was used to determine the RBM24-regulated specific exons. Upper panel, Exon^+^ is exon inclusion and Exon^−^ is exon exclusion. Bottom panel, Exon^+^ (%) = Exon^+^ / the sum of Exon^+^ and Exon^−^. The data in each group were based on the analysis of 5–9 independent experiments from 5–9 mice and expressed as means ± SEM. *P < 0.05, **P ≤ 0.01. See also Table S1

In aggregate, we identified 590 (belong to 292 genes) altered splicing events (Table S1). Five basic and generally recognized AS modes were investigated in our study, including skipped exons (SE), mutually exclusion exons (MXE), alternative 5′ splice sites (A5SS), alternative 3′ splice sites (A3SS), and retained intron (RI) (Fig. 3A). Interestingly, more than 70% splicing events in response to RBM24 ablation belonged to SE type and most of them were exon exclusion (Fig. 3B, Table S1), suggesting that RBM24 is a splicing repressor, consistent with previous studies (Yang et al., 2014; Zhang et al., 2016).

To obtain a functional overview of RBM24 regulated-AS transcripts, we grouped them into gene ontology (GO) analysis. As shown in Figure 3C (left), the analysis pointed to processes involved in cardiac functions, such as cytoskeleton organization, striated muscle cell differentiation, heart contraction, cardiovascular system development, and blood circulation. Subcategories of molecular function were also shown in Fig. 3C (right).

Importantly, the global AS analysis revealed that RBM24 regulated a network of genes previously reported to associate with heart diseases (Fig. 3D). *ABCC9*, which encodes for a potassium channel subunit, is mutated in human DCM (Bienengraeber et al., 2004). TTN (Liu et al., 2017a, b), NEBL (Purevjav et al., 2010) and FHOD3 (Arimura et al., 2013) are sarcomeric proteins which were reported to have DCM-associated variants. ENAH/VASP (Benz et al., 2013) and MFN2 (Ong et al., 2017) knockout mice could produce a final DCM phenotype. In addition, several RBM24-regulated alternative spliced transcripts have been previously linked to hypertropic cardiomyopathy, including *Slc25a3* (the mitochondrial phosphate carrier) (Mayr et al., 2007) and *Map4* (involved in microtubule network) (Cheng et al., 2010). We validated above AS events using RT-PCR (Fig. 3E). Results showed that RBM24 knockout changed the expression of alternatively spliced exons in all cases, indicating RBM24 regulated alternative isoform switch of each detected gene.

### Misregulated AS of contractile genes leads to sarcomere disarray in RBM24 deficient heart

**Figure 4:**
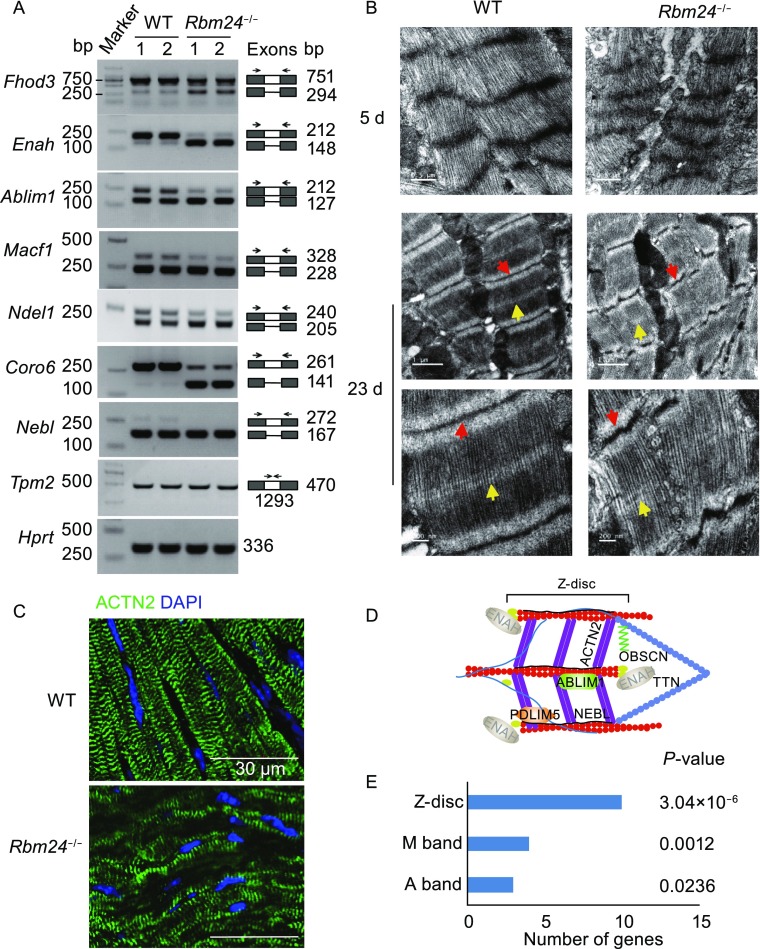
**Misregulated sarcomeric AS events result in disruptive sarcomere structure in RBM24 knockout mouse**. (A) Splicing analysis for RNAs related to sarcomere and cytoskeleton. Primer locations and expected band sizes are indicated. *Hprt* was used as an internal control. Data are representative of 5–9 independent experiments from 5–9 mice. (B) Representative electron microscopy analysis of sarcomere structure in *Rbm24*^−/−^ and WT mice at days 5 and 23. The red and yellow arrows indicate Z-disc and M-band, respectively. (C) Immunofluorescence imaging of sarcomeric protein ACTN2 (green fluorescence) in WT and *Rbm24*^−/−^ mice. Scale bar: 30 μm. Data are representative from 5–7 mice (B and C). (D) Schematic of cytoskeletal protein complexes within the cardiomyocyte Z-disc. Proteins located on the Z-disc are depicted. (E) GO analysis of sarcomere genes AS regulated by RBM24. See also Fig. S2

**Figure 5:**
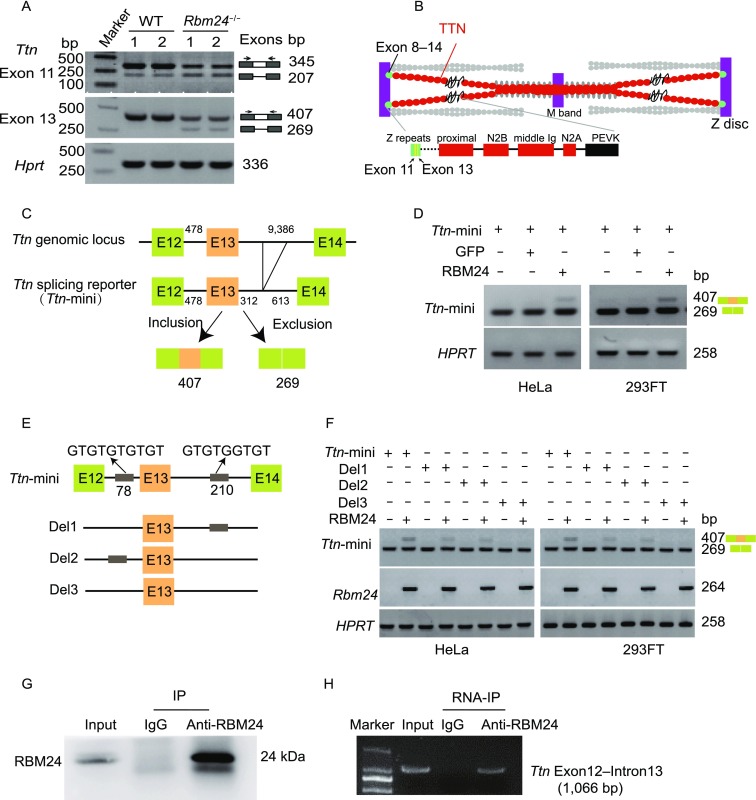
**RBM24 regulates** ***Ttn*** **splicing**. (A) Splicing analysis for *Ttn*. Primer locations and expected band sizes are indicated. Data are representative of 5–7 independent experiments from 5–7 mice. (B) A schematic of TTN structure. A single TTN molecule spans from Z-disc to the M-band. The structure annotation of TTN is indicated based on Roberts et al. (2015) and Anderson and Granzier (2012). (C) Schematic of *Ttn* splicing reporter (*Ttn*-mini) constructed based on *Ttn* genomic locus. The lengths of exons and introns are indicated. (D) RT-PCR analysis of the splicing pattern of *Ttn*-mini after transfection into HeLa and 293FT cells. (E) Schematic of exon 13 and the flanking introns of *Ttn*-mini. Two clusters of GT stretches located in introns upstream (cluster 1) and downstream (cluster 2) of exon 13 are indicated. Schematic of constructs with deletion of cluster 1 (Del1), cluster 2 (Del2) or both (Del3) from the *Ttn*-mini reporter are also shown. (F) RT-PCR analysis of RNA splicing in HeLa and 293FT cells transfected with the indicated plasmids. (G) Western blot analysis for RNA-IP experiments using anti-RBM24 antibody. RNA-IP was performed using anti-RBM24 antibody or a IgG negative control. (H) The enrichment of *Ttn* pre-mRNA showed in our experiment. RT-PCR analysis of RNAs isolated in RNA IP experiment in (G). RNA was reverse transcribed and quantified with RT-PCR using *Ttn* specific primers designed for 1,066 bp amplicon from exon12–intron13 region. *Hprt* was used as an internal control (A, D and F)

Failure to provide sufficient contraction is the hallmark of DCM and heart failure, and is often associated with defects in the assembly of the contractile apparatus (Tayal et al., 2017). Therefore, we subjected sarcomere (*Fhod3*, *Enah*, *Ablim1*, *Nebl*, *Tpm2* and *Ttn*) and cytoskeleton organization (*Macf1*, *Ndel1* and *Coro6*) related genes to a more detailed analysis. As shown in Figure 4A and 5A, in all cases we observed aberrant AS in *Rbm24*^−/−^ hearts, suggesting RBM24 regulated sarcomeric assembly and heart contractility through splicing. We further substantiated this observation using alternative cell line of mouse cardiac HL-1 after RBM24 knockdown (Fig. S2). These mis-splicings in the cardiac muscle contractile apparatus likely account for the abnormalities in heart morphogenesis and the dysfunction in cardiac contraction found in the *Rbm24*^−/−^ hearts.

Next, we performed ultrastructural transmission electron microscopy (TEM) to examine the sarcomere organization in the left ventricular tissues from *Rbm24*^−/−^ and WT mice. On day 5, the sarcomere structure of the *Rbm24*^−/−^ heart showed relatively minor defects (Fig. 4B). However, striking alterations in the myocardial structure of *Rbm24*^−/−^ hearts were evident on day 23, including less, substantially short and dramatically disarrayed sarcomere, irregular and wavy Z-disc (red arrows in *Rbm24*^−/−^, Fig. 4B) and M-band (yellow arrows in *Rbm24*^−/−^, Fig. 4B). Immunofluorescence also showed that the distribution of the Z-disc, labeled with anti-ACTN2 antibody, was affected in *Rbm24*^−/−^ mice (Fig. 4C). These changes might result from the altered isoform expression of proteins that were located at Z-disc (Fig. 4D). Consistent with the morphology analysis, GO analysis shows that most of genes spliced by RBM24 are located at Z-disc (Fig. 4E). These Z-disc genes are involved in maintaining the sarcomere structure and provide an anchoring site for filaments, which are important for the force generated by contraction. Defects in the components of Z-disc can trigger cardiac disease pathways, such as DCM (Knöll et al., 2002).

### RBM24 directly regulates *Ttn* splicing

*TTN* is the most frequently mutated gene in human DCM (Weintraub et al., 2017). Noted that RBM24-dependent gene network includes *Ttn*, we examined *Ttn*’s splicing by RT-PCR. Result showed that RBM24 promoted the inclusion of *Ttn* exons 11 and 13 (Fig. 5A), which are located in the Z-disc (Fig. 5B) and participates in myofibril assembly, stabilization, and maintenance (Roberts et al., 2015).

To confirm whether RBM24 is a direct splicing factor of *Ttn*, we then further analyzed the specific activity of RBM24 on *Ttn* RNA using *Ttn* splicing reporter (*Ttn*-mini) (Fig. 5C). With this reporter system, splicing regulation could be monitored from a single reporter vector that expressed a 407 bp fragment or a 269 bp fragment in case of exon 13 exclusion. As shown in Figure 5D, transfection of RBM24 into HeLa or 293FT cells led to the inclusion of exon 13. Thus, expression of RBM24 alone is sufficient to drive the AS of *Ttn* in cardiac and non-cardiac cells, and this splicing evidently does not rely on a tissue-specific cofactor.

We next attempted to determine whether RBM24-binding motif is required for RBM24-mediated AS. Previous study based on high-throughput *in vitro* binding assay suggested that RBM24 bound to G(A/U)GUG motif (Ray et al., 2013). We found two clusters of GT stretches in the *Ttn* gene that were located in introns upstream and downstream of exon 13 (Fig. 5E). Cluster 1 is located 78 bp upstream of exon 13. Cluster 2 resides 210 bp downstream of exon 13. To test the function of these two clusters for RBM24-mediated AS, we deleted cluster 1 (Del1), cluster 2 (Del2) or both (Del3) from the *Ttn*-mini reporter (Fig. 5E). Transfection into 293FT and HeLa cells revealed that either one of the clusters, upstream or downstream, was sufficient for RBM24-dependent inclusion of exon 13 (Fig. 5F). Subsequently, RNA immunoprecipitation (RNA-IP) was performed to determine whether RBM24 could directly bind to *Ttn* pre-mRNA. Western blot verified the expression of RBM24 in immunoprecipitated samples (Fig. 5G). In immunoprecipitates, a 1,066-bp amplicon of *Ttn* was found (Fig. 5H), indicating that RBM24 could directly bind pre-mRNA of *Ttn*.

Taken together, out data demonstrated that *Ttn* is the direct splicing target of RBM24, and the knockout of RBM24 resulted in aberrant splicing of *Ttn* and contributed to disarray of sarcomeric structures in the cardiomyocytes, a typical feature of DCM. This finding is similar to the other cardiac RNA binding protein RBM20, the only known splicing factor of *TTN* (Guo et al., 2012).

## Discussion

**Figure 6:**
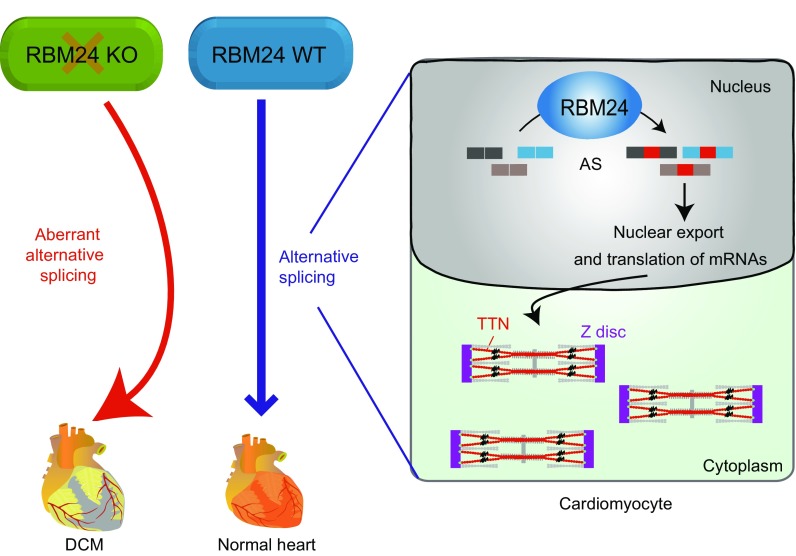
**A working model of RBM24 as a key splicing regulator in postnatal heart remodeling**. Tissue-specific ablation of RBM24 in the heart disrupts global AS and causes lethal DCM

All tissues examined have revealed the importance of tissue-specific RBPs in the regulation of complex networks involved in organ morphogenesis, maturation, and function (Blech-Hermoni and Ladd, 2013). RBM24 is essential for early cardiac development as a key splicing regulator (Yang et al., 2014; Zhang et al., 2016). However, postnatal heart is accompanied by major splicing changes (Cooper, 2005). We here performed a systematic analysis of postnatal function of RBM24 and presented RBM24 as a critical player that orchestrated isoform transitions of a network of genes with essential cardiac functions in postnatal development and diseases. A proposed working model is shown in Fig. 6. Our results highlight the distinct regulatory network for RBM24 function at different developmental stages, providing an interesting insight into the mechanism by which RBP regulates splicing in a context-dependent manner.

Our study identified more than 500 misregulated isoform switches in response to RBM24 deficiency, the majority of them were not captured in the previous studies focusing on early cardiogenesis (Yang et al., 2014; Zhang et al., 2016) (Fig. S3). Notably, a subset of them were reported to be associated with cardiomyopathy. RBM24 deletion resulted in the missplicing of a subset of sarcomere structure proteins, such as *Tpm2*, *Ttn*, *Nebl*, *Fhod3*, *Enah* and *Ablim1*, which is in accordance with the disruptive sarcomere in *Rbm24*^−/−^ heart. Collectively, our data suggests a model in which RBM24 deficiency results in altered isoform expression of genes. These aberrant isoform switches may result in altered biomechanics and signaling pathways that ultimately lead to impaired contraction, heart failure and postnatal lethality.

To date, only one RBP RBM20 has been identified as a splicing factor of *TTN* (Guo et al., 2012). Intriguingly, we discovered *Ttn* was the splicing target of RBM24 in this study. The giant TTN protein was found in approximately 25% of patients with DCM (Weintraub et al., 2017). Despite the established link between *TTN* mutation and DCM, the regulator that determines *TTN* splicing remains largely unknown. Only recently, a mechanistic understanding was reached when RBM20 was found to be involved in defective splicing of *Ttn* in a spontaneously occurring rat strain exhibiting symptoms of DCM (Guo et al., 2012). Our results showed that RBM24 promoted the inclusion of *Ttn* exons 11 and 13, which were located in the Z-disc and previously reported that they could bind to ACTN2 (Gregorio et al., 1998). Exclusion of these two exons causes Z-disc with less *Ttn* Z-repeats and hence less connecting ACTN2 and filaments (Gregorio et al., 1998). These might be a reason for the disruption of ACTN2 distribution in Z-disc (Fig. 4C). Thus, the loss of RBM24 may decrease the number and distribution of filaments in Z-disc, affect the Z-disc structure, and result in inadequate cardiac function in RBM24-deficient mice. Notably, besides *Ttn*, most of sarcomeric genes spliced by RBM24 were also located at Z-disc, such as *Nebl*, *Ablim1* and *Enah* (Fig. 4D and 4E), implying a regulatory role of RBM24 in establishing the mechanical coupling and the stretch-sensor mechanism of heart.

In conclusion, this study identifies a critical role of RBM24-mediated AS during postnatal heart development and pathogenesis. Our data not only offer mechanistic insights but also provide functional annotation of RBM24 splicing targets that contribute to DCM and heart failure, highlighting the key role of post-transcriptional regulation in physiological cardiac function and pathogenesis. It will be of great interest to determine in future studies whether the dysfunctional *RBM24* gene could be correlated to cardiac malfunction in humans, and potentially develop new therapeutic avenues for DCM disease.

## MATERIALS AND METHODS

### Ethics and animal care

All experimental protocols were approved by the Institutional Animal Care and Use Committee (IACUC) of Xiamen University (Xiamen, Fujian, China; approval ID: SCXK2013-0006). For cardiac function assay, mice were anaesthetized with inhalation of 5% isoflurane and maintained with 2% isoflurane (mice less than 10 days old did not have to be anesthetized). Mice were euthanized using CO_2_ inhalation and cervical dislocation. Death was confirmed by ascertaining cardiac and respiratory arrest.

### RBM24 cardiac-specific knockout mice

The detailed description of the generation of RBM24 cardiac-specific knockout mice is provided in the Supplementary Materials and Methods. To disrupt the *Rbm24* gene in mouse heart, we bred a mouse strain containing the *Rbm24* conditional alleles then crossed with mice carrying the Cre recombinase gene driven by the α-*Mhc* promoter (Wang et al., 2005). In this study, WT littermates (*Rbm24*^loxp/loxp^, *Rbm24*^loxp/WT^ or mice containing WT *Rbm24* allele) were used as controls and are referred to as WT throughout the text and Figures.

### Cardiac function assay

Echocardiography was performed to analyze cardiac function in mice. For details, please refer to the Supplementary Materials and Methods.

### RNA-seq and splicing analysis

Total RNA was isolated from littermate control hearts or *Rbm24*^−/−^ hearts (*n* = 2). For details, please refer to the Supplementary Materials and Methods.

### Generation of RBM24 knockdown HL-1 cells

AT-1 murine cardiomyocytes-derived HL-1 cells (gift from W. Claycomb, Louisiana State University) were maintained as previously described (Liu et al., 2017). HL-1 cells were infected with lentivirus expressing short hairpin RNA (shRNA) targeting mouse *Rbm24* gene (Santa Cruz Biotechnology) and selected with puromycin, nontargeting viral particles were used as a control.

### RNA immunoprecipitation and RIP-PCR

RNA immunoprecipitation and RIP-PCR was performed following the protocol described previously (Lin et al., 2017) using mouse heart lysates. Details are provided in the Supplementary Materials and Methods.

### Statistical analysis

Results were presented as mean ± SEM. For comparison between two groups, differences were analyzed by 2-tailed Student’s *t*-test. Differences were considered significant at a value of *P* < 0.05.

## Electronic supplementary material

Below is the link to the electronic supplementary material.

Supplementary material 1 (PDF 135 kb)

Supplementary material 2 (XLSX 208 kb)

Supplementary material 3 (XLSX 9 kb)

Supplementary material 4 (MP4 10510 kb)

Supplementary material 5 (MP4 9149 kb)

## Abbreviations

A3SS: alternative 3′ splice sites
A5SS: alternative 5′ splice sites
AS: alternative splicing
DCM: dilated cardiomyopathy
EF: ejection fraction
ESC: embryonic stem cell
FS: fractional shortening
GO: gene ontology
IVSd: interventricular septal thickness at end diastole
IVSs: interventricular septal thickness at end systole
LVIDd: left ventricular internal diameter at end diastole
LVIDs: left ventricular internal diameter at end systole
LVPWd: left ventricular posterior wall thickness at end diastole
LVPWs: left ventricular posterior wall thickness at end systole
LV Vold: left ventricular volume at end diastole
LV Vols: left ventricular volume at end systole
MXE: mutually exclusion exons
RBM24: RNA binding motif protein 24
RBP: RNA binding protein
RI: retained intron
RNA-IP: RNA immunoprecipitation
RNA-seq: RNA sequencing
SE: skipped exons
