# Supplementary material for: RNA binding protein 24 deletion disrupts global alternative splicing and causes dilated cardiomyopathy
Source: Protein Cell. 2018 Sep 28;10(6):405–16. doi: 10.1007/s13238-018-0578-8 (PMC6538757; doi:10.1007/s13238-018-0578-8)
Supplement: Supplementary file 1 — Supplementary material 1 (PDF 135 kb) [file 13238_2018_578_MOESM1_ESM.pdf]

Supplementary Information

RNA binding protein 24 deletion disrupts global alternative splicing and causes dilated cardiomyopathy

Running title: Deletion of RBM24 in postnatal heart causes DCM

Keywords: RNA binding protein; RBM24; dilated cardiomyopathy; alternative splicing; heart failure;

Jing Liu[1¶], Xu Kong[1¶], Meng Kai Zhang[1], Xiao Yang[2], Xiu Qin Xu[1]*

[1] The Institute of Stem Cell and Regenerative Medicine, Medical College, Xiamen University, Chengzhi Building, Xiang'an Campus, Xiamen, Fujian Province, 361100, P.R. China

[2] State Key Laboratory of Proteomics, Genetic Laboratory of Development and Disease, Institute of Biotechnology, 20 Dongdajie, Beijing, 100071, P.R. China.

* Correspondence: xuxq@xmu.edu.cn

[¶] These authors contributed equally to this work.

Inventory of Supplementary Information

Text S1. Supplementary information

Table S1. RBM24-dependent splicing events identified by RNA-Seq in RBM24 WT and -/- mice at 5 days old (Related to Fig. 3, supplied as separate excel file).

Table S2. Primers for AS assay, splicing reporter construction and RT-PCR (Supplied as separate excel file).

Video S1. Video of WT mice at 23 days (Related to Fig. 2).

Video S2. Video of -/- mice at 23 days (Related to Fig. 2).

Text S1. Supplementary information

Supplementary Materials and Methods

Generation of RBM24 cardiac-specific knockout mice.

The mouse *Rbm24* gene is encoded by four exons of genomic DNA. We constructed a targeting vector containing loxP sites (triangles) flanking exon 2 and exon 3 of the *Rbm24* gene (Fig. 1A). The neomycin (Neo) selection cassette was flanked by Flpase Recognition Target (FRT) sites, allowing for subsequent excision with Flpase. The vector was introduced into B6/BLU mouse embryonic stem cells (ESCs), and G-418-resistant transformants containing the mutation in one allele of the *Rbm24* gene were established. The ESC clones were aggregated with 8-cell embryos, and the embryos were implanted into foster mothers to obtain mice. Then mice were crossed with a Flper mice to remove the Neo selection cassette, then their offspring (*Rbm24*$^{\mathrm{loxp/WT}}$) were crossed to a transgenic mouse line in which the Cre recombinase was expressed under the control of alpha myosin heavy chain (α-*Mhc* promoter (*Mhc*cre), which directed cardiac-specific expression (Wang et al., 2005).

Genotyping on mouse-tail DNA was performed using a single PCR reaction to identify WT and RBM24 knockout alleles. Genomic DNA was used in a reaction that included two primers: the WT and conditional *Rbm24* allele were amplified using forward 5’-CATGGATGTTGGTGGTGCTG-3’ and reverse primer 5’-GACCTGGCGGTAGACAGACAT-3’ primers. Cre was amplified using forward 5’-ATTTGCCTGCATTACCGGTC-3’ and reverse primer 5’- ATCAACGTTTTCTTTTCGG-3’ primers. PCR was performed as follows: 32 cycles (each cycle consisting of 30 s at 95°C, 30 s at 56°C, and 50 s at 72°C) with an initial denaturation step at 95°C for 2 min, and a final elongation step at 72°C for 2 min.

Cardiac function assay

Mice were placed on a heated platform set at 37°C and anesthetized with isoflurane gas (mice less than 10 days old does not have to be anesthetized). Transthoracic measurements were taken using a Vevo-2100 Imaging System with a MS550 linear array transducer. M-mode images were acquired from the parasternal long-axis view at the papillary muscle level. Data analysis was performed with the use of the VevoStrainTM Analysis software.

RNA-seq and splicing assay

Total RNA was isolated from littermate control hearts or -/- hearts (n=2). RNA was extracted using Trizol regent, and RNA-seq library was prepared after rRNA depletion according to manufacturer's instruction (Illumina, http://www.illumina.com). The cDNA library was sequenced on the Illumina sequencing platform (Illumina HiSeq PE150). In total, 116.2 million (5 d WT: 59.4, 56.8) and 122.6 million (5 d -/-: 61.3, 61.3) reads were obtained and aligned to the mouse genome (GRCm38/mm10), and splice junctions were identified using TopHat. Uniquely mapped reads (75%-84% of sequenced reads) were selected for coverage and junction read count assembly. Replicate MATS (rMATS) was used to analyze AS (Shen et al., 2014). RNA-seq data were uploaded to the NCBI's BioProject database under accession numbers PRJNA415257.

Ultrastructural examination by transmission electron microscopy (TEM)

A portion of each heart sample was cut into 1×1×1 mm cubes with a surgical-grade blade and fixed immediately in 0.1 M PBS (pH 7.2) with 3% glutaraldehyde and 1.5% paraformaldehyde at 4°C for at least 2 h. Then, samples were rinsed with 0.1 M PBS (3 times), fixed with 1% osmium tetroxide and 1.5% kalium ferrocyanatum at 4°C for 2 h. After graded acetone dehydration, samples were saturated with anhydrous acetone and epoxy

resin embedding medium for 1.5 h, then embedded in pure embedding medium. Resultant samples were sectioned into ultra-thin sections (70-80 nm), stained with uranyl acetate and lead citrate, then observed using Tecnai G2 Spirit BioTwin TEM (FEI).

Immunofluorescence

Mice were sacrificed, hearts were isolated and embedded in OCT for immunofluorescence. 5 μm thick sections were cut and fixed in 4% paraformaldehyde for 15 min, then washed three times with PBS and incubated with anti-ACTN2 antibody (Sigma, A7811, 1: 800) for overnight at 4°C. After incubation with primary antibody, sections were rinsed with PBS three times, and then incubated for 1 h at room temperature in the dark with AlexaFluor® 488 conjugated goat anti-mouse IgG (H+L) secondary antibody (Invitrogen, 1: 500). Sections were subsequently washed in PBS three times (5 min), and cell nuclei were counterstained with Hoechst 33258 (Sigma, 1: 1000).

Histopathological analysis

Heart tissue was fixed in 4% paraformaldehyde, embedded in paraffin, and cut into 5 μm thick slides using microtome (Leica). Hematoxlyn and eosin (H&E) and Masson's trichrome stain were performed using kits from Jiancheng Bioengineering Institute.

RT-PCR and Real-time PCR

Total RNAs were extracted using Trizol (Invitrogen), followed by cDNA synthesis using SuperScript II reverse transcriptase (Invitrogen). 25-30 cycles for PCR were used. PCR products were analyzed by electrophoresis on high-resolution agarose gel. Real-time PCR was performed on an ABI 7500 thermal cycler in 20 μl reactions, with 10 nM of each primer and 2 μl of each cDNA preparation, using qPCR SYBR Green Mix (Promega) as per

manufacturer's instructions. Primers for RT-PCR and Real-time PCR were listed in Table S2.

Construction of splicing reporters

The *Ttn* mini-splicing reporter (*Ttn* -mini) recapitulates splicing of the *Ttn* genomic locus. *Ttn* intron 12 and 13 were shortened internally. *Ttn*-mini was assembled from 2 fragments: exon 12-intron 12-exon 13 and intron 13-exon 14. Then fragments were cloned into pcDNA3.1 (+) plasmid using the following restriction sites: KpnI and XhoI via pEASY-Uni Seamless Cloning and Assembly Kit from Transgen biotech. Primers for splicing reporter construction were listed in Table S2. For splice reporter assay, splicing reporters were transfected into Hela or 293FT cells using Lipofectamine 2000 (Invitrogen) according to manufacturer's instruction. After 48 hours, total RNA was isolated, and RT-PCR was used to examine splicing pattern.

Cells culture and Transfection

HeLa and 293FT cells were cultured in DMEM (Gibco) supplemented with 10% fetal bovine serum (Gemini) and penicillin/streptomycin at 37℃/5% CO_2 in an incubator. Transfections were performed using Lipofectamine 2000 (Invitrogen) according to the manufacturer's protocol.

RNA immunoprecipitation and RIP-PCR

Mouse hearts were isolated and lysed by polysome lysis buffer (PLB) with RNase and protease inhibitors. In order to reduce the background, the lysate was incubated overnight with protein G agarose beads (Millipore) at 4°C for pre-incubation. The following day, the supernatant was immunoprecipitated by anti-RBM24 antibody beads. After

immunoprecipitation, Bead-bound complexes were washed 3 times, and RNAs were extracted using Trizol. Then RT-PCR was performed for RIP-PCR using forward 5'-ATGATGAAGGAAACTAGGAAAAC-3' and reverse primer 5'-CGTTGAGTCTCTTTGATATTAGCC-3' primers.

Western blotting

Proteins from mouse heart were harvested with RIPA lysis buffer supplemented with protease inhibitors. Then, proteins were separated on SDS-PAGE gels, transferred to PVDF membrane, and detected by enhanced chemiluminescence. The following primary antibodies were used: anti-RBM24 antibody (Abcam, 1: 1000) and anti-GAPDH antibody (Transgen, HC301, 1: 5000).

Supplementary Results

Comparison of studies on RBM24-regulated alternative splicing events

To compare our results with previous study on RBM24-regulated AS events, we evaluated our list of 292 genes (Table S1) with RBM24-regulated genes presented by Yang *et al*. (Yang et al., 2014). The study by Yang *et al.* profiled -/- embryonic hearts and identified 68 genes that regulated by RBM24. Among these genes, 31 overlapped with ours (Fig. S3). Notably, we observed that many RBM24-regulated AS events related to diseases such as *Ttn*, *Nebl, Fhod3*, and *Mfn2* were not found in Yang *et al.*'s study. The remolding of transcription level during postnatal development might account for the changes in AS. A number of additional RBM24-regulated genes identified in this study is discovered the postnatal heart. We should keep in mind that a direct comparison between different RNA-seq studies is often difficult. For example, the list of 68 genes assembled by Yang *et al*. was only a selection of RBM24-regulated AS events based on embryonic stage. The limited intersection of cardiac enriched genes might further be explained by technical limitations associated with cross-platform comparison (Yang *et al*.: Illumina HiSeq PE100, aligned to the mm9 vs our study: Illumina HiSeq PE150, aligned to the mm10). It also highlights the need for independent profiling approaches and utilizing RBM24 -/- mice at different developmental stages.

Supplementary Figures

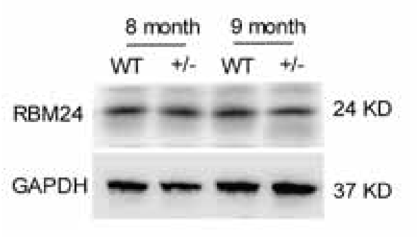

Figure. S1 Western blot analysis of RBM24 protein from WT or +/- mice at different ages. GAPDH served as a loading control. Data in each group are representative of 5-10 mice

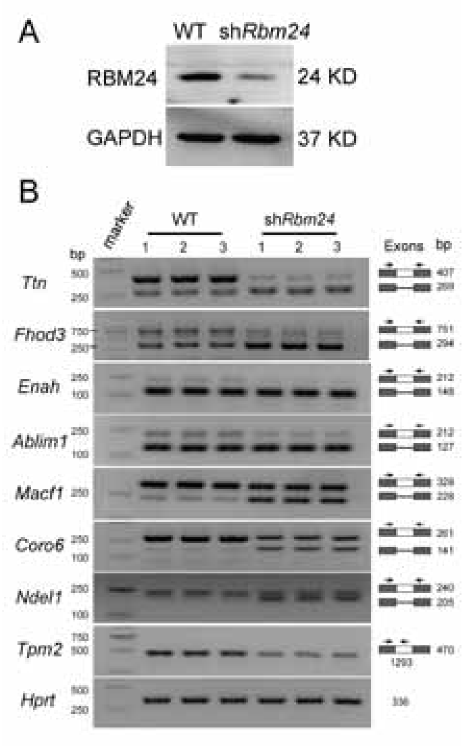

Figure. S2 Misregulated sarcomeric AS events in HL-1 cells after RBM24 knockdown. (A) HL-1 transfected with control vector or sh*Rbm24*. Western blot was done using antibodies against RBM24 and GAPDH. (B) Splicing analysis for RNAs related to sarcomere and cytoskeleton. Primer locations and expected band sizes are indicated. *Hprt* was used as an internal control. Data are representative of 5 independent experiments.

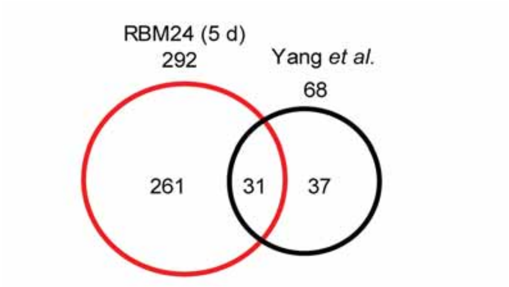

Figure. S3 Comparison of studies on RBM24-regulated AS events. Venn diagram showing the overlap of splicing genes between ours and Yang *et al*.

References

Shen S, Park JW, Lu ZX et al. (2014) rMATS: robust and flexible detection of differential alternative splicing from replicate RNA-Seq data. Proc Natl Acad Sci U S A 111(51): E5593-5601.

Wang J, Xu N, Feng X et al. (2005) Targeted disruption of Smad4 in cardiomyocytes results in cardiac hypertrophy and heart failure. Circ Res 97(8): 821-828.

Yang J, Hung LH, Licht T et al. (2014) RBM24 is a major regulator of muscle-specific alternative splicing. Dev Cell 31(1): 87-99.
